# Transcatheter Closure of Perimembranous Ventricular Septal Defects Using Different Generations of Amplatzer Devices: Multicenter Experience

**DOI:** 10.1155/2020/8948249

**Published:** 2020-02-21

**Authors:** Roberto Mijangos-Vázquez, Amal El-Sisi, Juan P. Sandoval Jones, José A. García-Montes, Rogelio Hernández-Reyes, Rodina Sobhy, Antoine Abdelmassih, Mohammed M. Soliman, Safaa Ali, Tatiana Molina-Sánchez, Carlos Zabal

**Affiliations:** ^1^Pediatric Interventional Cardiology Department, Pediatric Specialties Hospital, Tuxtla Gutiérrez, Chiapas, Mexico; ^2^Pediatric Department of Faculty of Medicine, Cairo University Children Hospital, Cairo, Egypt; ^3^Pediatric Interventional Cardiology Department, National Institute of Cardiology “Ignacio Chávez”, Mexico City, Mexico; ^4^Pediatric Department of Faculty of Medicine, Sohag University Hospital, Sohag, Egypt

## Abstract

**Objectives:**

To demonstrate safety and efficacy of using different generations of softer Amplatzer™ devices for ventricular septal defect (VSD) closure to avoid serious complications at follow-up.

**Background:**

Transcatheter closure of perimembranous ventricular septal defects (PmVSD) is a well-established procedure; however, it is associated with unacceptable incidence of complete heart block. Great advantages have been achieved by using softer devices for VSD transcatheter closure. The first and second generation of Amplatzer™ occluders (AVP II, ADO, and ADO II) seem to offer a safe and attractive alternative for this procedure. These devices can be delivered using either an arterial (retrograde) or venous (prograde) approach.

**Methods and Results:**

Patients with congenital PmVSD who underwent transcatheter closure using ADO, ADO II, and AVP II devices were included. Primary end point was to determine efficacy and safety of these generations of devices and to determine the incidence of complications at follow-up (complete AV block and aortic/tricuspid/mitral regurgitation). One hundred and nineteen patients underwent VSD closure at a median age of 5 years (8 months–54 years). During the catheterization, there were only minor complications and at follow-up of 36 ± 25.7 months (up to 99 months), the closure rate was high of 98.3% and freedom from AV block was 100%.

**Conclusions:**

The use of softer Amplatzer™ devices is a good alternative to achieve PmVSD closure safely with no risk of AVB during the procedure or at midterm follow-up.

## 1. Introduction

Ventricular septal defects (VSD) are the most common congenital heart malformation accounting for almost one-fifth of all defects and can be located in membranous or muscular septum. Eighty percent of these are perimembranous [1].

Surgical closure has been regarded as the gold standard treatment. However, transcatheter closure of congenital VSD for specific and selected patients has been advocated as an alternative to surgical repair as it avoids the risks of cardiopulmonary bypass, postoperative discomfort, and the need for sternotomy and a residual scar [2, 3].

Percutaneous closure of VSD was first described by Lock et al. [4] in 1988, when devices designed for closure of patent ductus arteriosus (PDA) and atrial septal defects (ASD) were implanted in the interventricular septum with variable degrees of success [5, 6].

For a successful VSD closure, an anatomic delineation of the defect with its relation to other cardiac structures is needed, so that the development of new aortic/tricuspid regurgitation or conduction defects can be avoided [7]. The presence of an aneurysm in the membranous septum is a frequent finding [8], which is useful as the device disk could be accommodated within the aneurysmal sac. Pedra et al. [9] pointed out that depending on the anatomy, the device is positioned either completely in the aneurysm or with the left ventricular disk at the crest of the defect, minimizing interference with the tricuspid or aortic valves.

With the development of the self-centering Amplatzer™ devices, an increasing number of defects could be closed with specially designed occlusion devices [10, 11], including the Amplatzer™ Muscular Ventricular Septal Defect Occluder (AMVO, Abbott, Golden Valley, MN) and the Amplatzer™ perimembranous ventricular septal occluder (APMVSDO).

Initial attempts at percutaneous closure of PmVSD resulted in heart block in 3% to 5% of patients in some reports [12]. Greater complications were encountered in patients weighing <10 kg [13]. As a result, the initial PmVSD device was withdrawn from clinical trial, and today, there is no FDA-approved device for transcatheter closure of PmVSD. All the complications caused the search of how to achieve VSD transcatheter closure with minor difficulties using other available devices such as the Amplatzer™ Vascular Plug II (AVP II) and Amplatzer™ Duct Occluder first and second generations (ADO and ADO II), (Abbott, Plymouth, MN, USA) in an off-label manner. Duct occluders for the closure of PmVSD were initially reported by Hieu [14] in 2002. These devices are softer because they have no occlusive fabric and can be delivered easily either prograde or retrograde through relatively small catheters. Theoretically, these devices, thus less likely to cause heart block, are softer than the previously tested devices. Additional advantage could be the lack of centering mechanism in the AVP II, which makes the device more mobile, further decreasing the possibility of pressure on the AV node or His bundle and the small central soft disc in ADO II. These devices are different from the APMVSDO which “stents” the defect, putting pressure along the rim of the VSD where the conduction system runs, thus resulting in heart block.

## 2. Methods

### 2.1. Patients

A retrospective study was conducted at 4 cardiac centers of all patients who underwent perimembranous ventricular septal defects closure with different generations of Amplatzer devices (ADO, ADO II, and AVP II). Institutional review board approval was obtained. Procedures performed from January 2011 through June 2019 were included. Patients were included if they [1] had perimembranous congenital ventricular septal defects; [2] had left ventricle (LV) dilation (Haycock, Z-sore >2); and [3] had VSD closure using generation I and II of the Amplatzer™ Duct Occluder (ADO I and II) devices or Amplatzer™ Vascular Plug II (AVP II) device. It is important to comment that most of the patients had only mild LV dilation. Patients were excluded if they [1] had residual defects after surgery; [2] had postinfarction VSD; [3] had VSD closure with different devices from the ones mentioned before; and [4] had association with infective endocarditis during the time of patient selection. For device selection, we measured the narrowest portion of the VSD based on measurement of the right ventricular side of the defect during left ventricle angiogram, and in case of selecting ADO device, it was chosen not to exceed this diameter by 2 mm; and in case of using ADO II, the central disc was chosen not to exceed this measure by 1 mm. In case of selecting AVP II, we chose not to exceed 2 mm at the LV defect size measured by fluoroscopy in patients with aneurysmal defects. In cases with aneurysmal PmVSD, we preferred ADO I or AVP II devices, and in tubular defects, we preferred the ADO II device. We considered a procedural success to the ability of deploying the device without embolization; new onset valvular regurgitation or increase in valvular insufficiency (if it was present before VSD closure); or the presence of complete heart block.

### 2.2. Cardiac Catheterization

**Figure 1:**
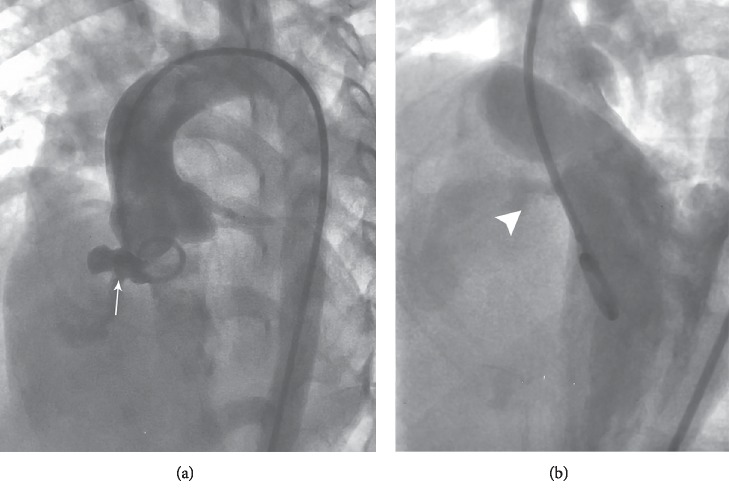
Initial four chambers angiography. (a) The arrow shows aneurysmal membranous VSD. (b) The arrowhead shows VSD. VSD = Ventricular septal defect.

**Figure 2:**
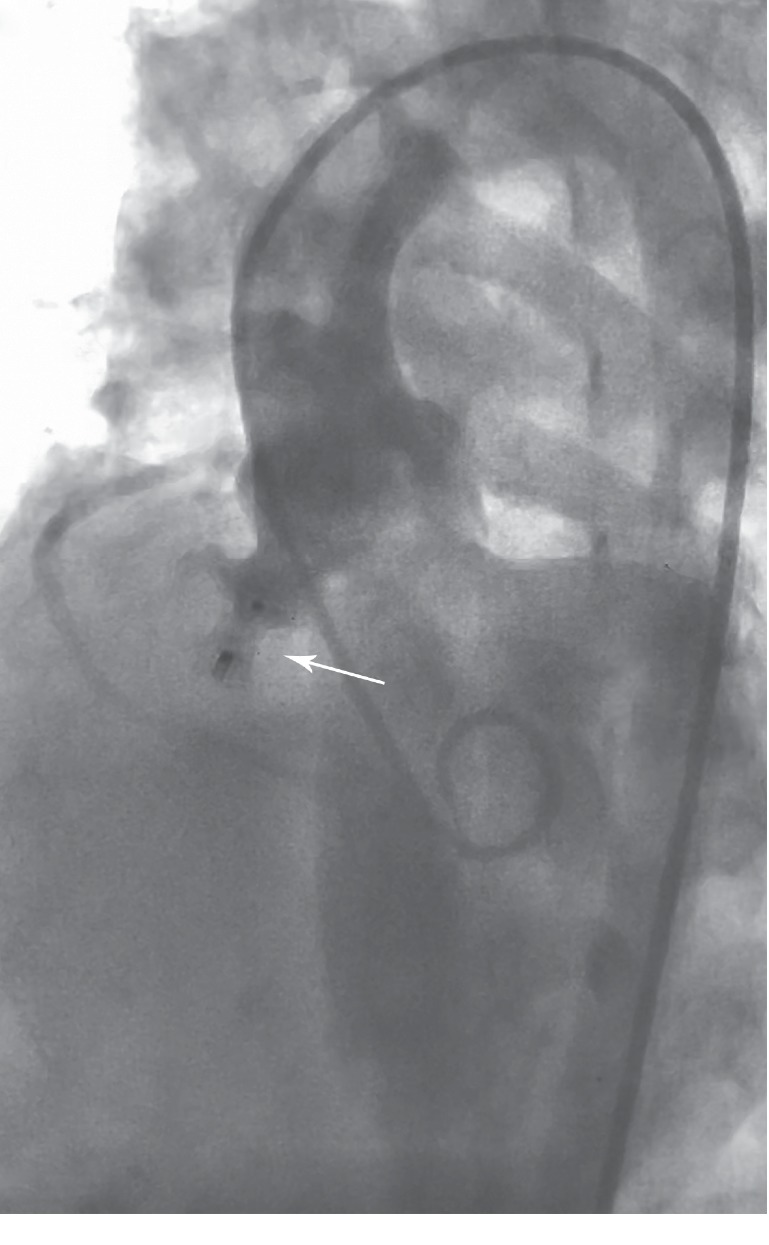
Four chambers fluoroscopy, once the ADO device has been placed inside the aneurysmal sac (arrow) by the prograde approach. ADO = Amplatzer duct occluder.

An informed consent form was given to the relatives of the patients, and a complete left and right heart catheterization with exclusive femoral arterial access in procedures with the retrograde approach and femoral arterial and venous access for the arteriovenous loop in procedures with the prograde approach were performed. All procedures were performed under general anesthesia. VSD was thoroughly investigated with left ventricular angiography in four chambers view (Figure 1); transesophageal echocardiogram (TEE) was used in 15 patients and and transthoracic echocardiogram (TTE) only in 2 patients; in most of our patients, we do not use echo guidance. In patients with prograde approach (62 patients), with the assistance of 0.035 inch and 260 cm hydrophilic coated Terumo™ guidewire and a 5Fr Judkins right (Cordis™, J&J) catheter, the defect was crossed from the LV to the right ventricle (RV) and then into the pulmonary artery (PA), superior vena cava (SVC), or inferior vena cava (IVC) based on the wire and catheter feasibility. After doing an AV loop, through the femoral vein, a delivery sheath was advanced and placed in the LV apex or in the ascending aorta (depending on the operator). ADO was loaded on the delivery system and advanced to the tip of the delivery sheath. Under fluoroscopy, the retention disk was deployed in the LV; then, the sheath and device were pulled slowly, and the device was deployed just as if closing a PDA (Figure 2).

**Figure 3:**
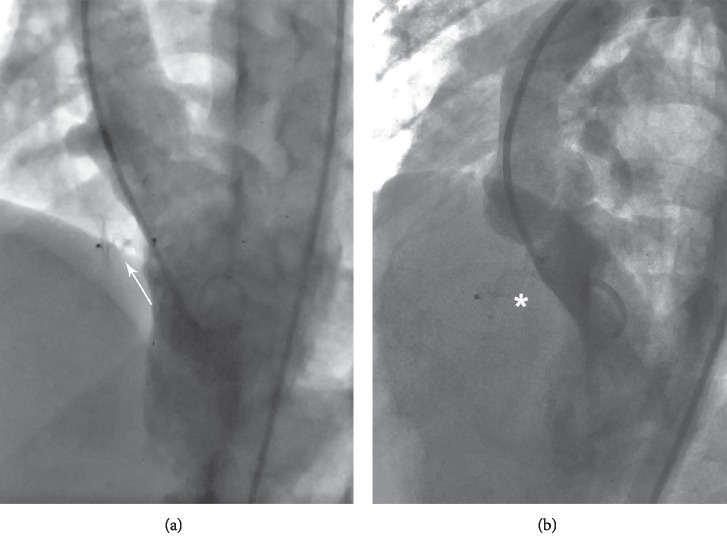
Four chambers fluoroscopy (a) once the ADO II device has been placed by the retrograde approach. (b) Arrowhead showing proper position of the AVP II with no residual shunt. ADO II = Amplatzer duct occluder II. AVP II = Amplatzer vascular plug II.

In the patients with retrograde approach (58 cases), after having crossed the VSD from the femoral artery, the hydrophilic coated Terumo™ guidewire was positioned in the RV or PA, and then a Terumo™ 5Fr Judkins right guiding catheter was advanced over the 0.035 inch Terumo™ guidewire into the RV. AVP II or ADO II was loaded on the guiding catheter and advanced to the tip of the catheter. Under fluoroscopy, the distal disk of the device was deployed in the RV; then, the catheter and device were pulled slowly, and the waist and the proximal disk of the device were deployed just in the LV side of the defect (Figure 3).

Proper position and residual shunt were confirmed with LV angiography and with TEE or TTE in some cases. After achieving good position of the device, we evaluated for any evidence of regurgitation of the aortic valve with angiography at the aortic root, and finally, device was released, and LV angiography was repeated. Next morning, electrocardiogram and TTE were performed.

Prophylactic antibiotics were used previous to the procedure, and intravenous heparin was used during the whole procedure at a dose of 100 U/Kg (maximum dose of 5000 UI).

### 2.3. Follow-Up

Patients left the hospital 24 hours after the procedure with prophylactic antibiotics and aspirin at a dose of 5 mg/kg (maximum dose of 100 mg) for 6 months. Follow-up visits at 1, 3, 6, and 12 months and then yearly to perform clinical examination, electrocardiogram, and a TTE. We chose this scheme of visits for the purpose of assessing the position of the device, possible residual shunt, presence of aortic, tricuspid, or mitral regurgitation, and to document cardiac rhythm.

### 2.4. Statistical Analysis

Frequencies and percentages expressed categorical variables. Continuous variables were summarized as mean (SD) or median (range) as appropriate, depending on normally of distribution. The statistical analysis was performed using the SPSS version 19.0 and Microsoft Excel 2016 for Mac.

## 3. Results

A total of 119 patients who met the inclusion criteria, were treated by catheterization at 4 cardiac centers (México and Egypt). In all the procedures for transcatheter PmVSD closure, ADO I was attempted in 55 patients; ADO II in 51 patients; and AVP II in 12 patients. The prograde approach was used in 62 patients and the retrograde approach in 58 patients; it was chosen depending on the anatomy of the defect. From the complete group of studied patients, 88 patients (74%) had aneurysmal defects and 31 patients (26%) had just the PmVSD with no aneurysm. A total of 10 patients had aneurysmal defects with multiple exit sites. This group included 62 female patients (52.5%) with a group median age of 5 years (ranged from 8 months to 54 years) and weight of 18 kg (6.5–87). Demographic data are shown in Table 1. It is important to mention that all patients included were intented to treat.

The mean systolic pulmonary arterial pressure was of 31.7 ± 7.9 mmHg. The mean of the pulmonary and systemic flow (*Q*_*p*_/*Q*_*s*_) was of 1.8 ± 0.8, and the mean diameter of the septal defect was of 4.9 ± 1.5 mm.

The devices used in all the patients are listed in Table 1. Previous to the procedure, a total of 4 patients had prolapse of the aortic cusp with no aortic regurgitation; one patient had aortic insufficiency and 6 patients mild mitral insufficiency as well; regarding the tricuspid valve function, it was completely normal in all treated patients. After devices were implanted, we found 16 patients (13.5%) with trivial residual shunt across the device evidenced by the angiography during the procedure. In one patient, the device (ADO 12/10 mm) slipped through the defect possibly due to tear of the ventricular septal aneurysm, and the device was still attached to the cable. The procedure was aborted, and the patient went to surgery. One patient developed transient complete heart block with normalization to sinus rhythm after treatment with steroids. All other patients had successful device implantation. In some of our patients, we found association with other cardiac defects, so we performed successfully during the same procedure, PDA closure in 4 patients, ASD closure in two, and PFO closure in one patient. No increase of previous aortic valve regurgitation was evidenced by pulling the guiding catheter to the aortic root and performing an angiography in that site before and after releasing the device. We decided to use TEE or TTE only in patients with high risk for the presence of previous aortic cusp prolapse and aortic or mitral valve regurgitation, and no increase of previous aortic or mitral valve regurgitation was evidenced. Tricuspid valve competence was evaluated by TTE 24 hours after the procedure.

Median fluoroscopy time was 27.3 minutes (range 7.2–61), and the median procedure time was 80 minutes (range 25–210). All patients were discharged 24 hours after the procedure. At a mean follow-up of 36 ± 25.7 months, 115 patients showed complete closure of the defect and 3 patients had trivial residual shunt across the device. A patient in whom we used an ADO I 10/8 mm device presented late embolization of the device to the right pulmonary artery (RPA) discovered by TTE examination 3 months after procedure, and he went to surgery. No AV block was found in electrocardiogram, and no increase in previous aortic, tricuspid, or mitral regurgitation was found by TTE.

## 4. Discussion

The VSD is the most common congenital heart disease, comprising 10% of all forms of congenital cardiac defects [15]. The percutaneous closure of the VSD has emerged as an important alternative to surgical repair. Initially, the success rates were not high, and the complication rate (complete AV block) was a very important limitation.

At the end of the 90s and the beginning of the 21^st^ century, the availability of Amplatzer™ VSD occluders, which was specially designed for occluding muscular and PmVSD, improved the success rate of this kind of procedures in regard to its safety profile and occlusion rates [16, 17]. However, most ventricular defects have deficient rim which would not allow the placement of the device disk without aortic valve distortion.

A conventional VSD closure technique involves complex steps. Occasionally, the delivery sheath is difficult to pass through a small defect or be positioned into the LV apex. These difficulties can make a difficult and challenging procedure with the conventional AMVO. Moreover, PmVSD closure using the APMVSDO device has largely been abandoned because of the risk of AV block. In a review published in 2005 by Carminati et al. [18] where 122 patients were catheterized, there was a 2.5% risk of heart block. In the follow-up, delayed complete heart block occurred in 2 patients with PmVSD, for which permanent pacemaker was inserted.

Since this early experience, many authors have published case series data on VSD closure using different generations of smaller Amplatzer™ devices in offlabel application. Behnke et al. [19] published a 12 patient series with membranous and muscular defects, most of them being infants. By example, they used an ADO 6/4 for a tunnel-like VSD in a small 4.4 kg baby successfully at a time when the membranous VSD devices were not available. In a study period of 79 months, Dilawar and Ahmad [20] published a total of 7 cases (6 perimembranous and 1 muscular) with tunnel-type aneurysmal tissue closed using 8/6 mm ADO. Diab et al. [21] recommended using the ADO in those patients where significant crowding at the right ventricle apex by muscle bundles prohibits the full expansion of the RV disc of the approved VSD occluders. Ebeid et al. [22] in a retrospective review and data, reported a total of 20 patients using the AVP II in 9 patients, and ADO II in 11 patients (10 delivered retrograde and 10 prograde). El Said et al. [23] in a retrospective analysis of 21 patients with PmVSD and aneurysm, reported 19 patients with successful ADO I implantation. Our series reports on PmVSD closure in 118 patients using ADO, ADO II, and AVP II devices with a high success rate of 98.3%.

For an appropriate selection of these percutaneous devices, Behnke [19] used 3–5 mm devices larger than the size of the defect. Ebeid et al. [22] suggested when the ADO II was used, the size of the center disc has to be equal to or 1 mm larger than the size of the color Doppler imaging. In aneurysmal defects, if AVP II was used, it was chosen to be 6-7 mm larger than the smallest VSD size by color Doppler, which would be equivalent to the ADO II retention disc size and will fix into the aneurism. Koneti et al. [24] suggest that the diameter of the ADO II is chosen to be either equal to or 1 mm greater than the smallest VSD diameter. El Said et al. [23] proposed three different locations for measurement of the defect: at the left ventricular opening of the VSD; at the largest diameter of the aneurysm, usually in the midsegment of the aneurysm; and at the right ventricular opening of the VSD aneurysm, usually the smallest diameter of the defect, where they proposed the use of a device 2 mm larger than this last measurement. Ghaderian et al. [25] proposed to select an ADO device at least 2 mm larger than the narrowest of the measured VSD diameter in right side by ventriculogram. As mentioned previously, we measured the narrowest portion of the VSD on the left ventricular side during the diastolic left ventricular angiogram, and the devices were chosen not to exceed this diameter by 2 mm in case of using ADO device; and in case of using ADO II, the central disc was chosen not to exceed this measure by 1 mm. In case of selecting AVP II, we chose not to exceed 2 mm at the LV defect size measured by fluoroscopy in patients with aneurysmal defects, which differs from that suggested by Ebeid et al. [22].

Percutaneous closure of PmVSD remains a real challenge; however, with the use of softer and smaller devices, we think that VSD closure can be more safe and effective. Dilawar et al. [20] informed that VSD closure rate was 100% (7/7) using ADO immediately after the procedure, and medium term follow-up of 6/7 patients revealed no residual VSD with 100% success. El-Sisi et al. [26] in a prospective observational single center study of 30 patients reported a 100% success using ADO devices (I and II). Ghaderian et al. [25] used an ADO device in 28 patients with a successful rate of 96.4%. Hua et al. [27] published a 100% successful closure of PmVSD in 16 patients using AVP II. Kanaan et al. [28] achieved a successful closure in 93.5% of 31 patients using ADO II. Zhao et al. [29] from China reported a success rate of the interventional procedure of 98% in 51 patients with PmVSD using ADO II device in a period of 4 years. Knop et al. [30] reported the closure of PmVSD in 6 patients using ADO II AS with no complications, but transitional complete right bundle branch block in only one patient resolved after a short course of steroid therapy. We achieved a high complete closure rate of 98.3% using these smaller devices (one patient presented slip of the device through the defect while the device was still attached to the cable, so we had to abort the procedure and sent him to surgery).

Using Amplatzer VSD devices, serious complications during and postprocedure have been presented. However, using these softer Amplatzer devices, only eventual and minor complications have been reported in regard to aortic, tricuspid, or mitral regurgitation. Dilawar [20] informed one patient who had progression of aortic valve regurgitation from trivial immediately after procedure to mild in a median of 54 months of follow-up. In the study of El Said et al. [23], new tricuspid regurgitation occurred in only 1 of 18 patients and was trivial. Tricuspid stenosis due to encroachment on the inflow of the tricuspid valve occurred in only one case, and it was mild. In our series, there were only minor postprocedure complications (slipped of the device through the defect which required to abort the procedure; and transient AV block, both in one patient each); and in the follow-up, there was no complete heart block or increase in aortic or mitral regurgitation. Only 3 patients (2.5%) presented with residual shunt and one patient with late embolization of the device to the RPA discovered by TTE 3 months after the procedure, possibly because of undersizing of the device. The patient went to surgery.

## 5. Conclusion

Use of ADO I, ADO II, and AVP II devices for closure of VSD seems to be safe and effective. They can be used as an offlabel therapy for VSD closure with good midterm outcomes. To date, they are preferable over devices designed exclusively for VSD closure.

### 5.1. Perspectives

It is known that the use of offlabel occluders for ventricular septal defect closure is becoming an emerging practice with high global success rate and encouraging outcomes. Now, this study demonstrates outstanding results in VSD closure referring to the use of softer devices with acceptable complication rate. However, there were certain limitations. Being a retrospective study, some of the obtained data were missing which reduce the impact of this work. In regard to the complications at follow-up, complete heart block was not observed in any patient; furthermore, it is important to know that all the patients were evaluated only by conventional electrocardiogram. So, we know that it is very important to have holter data for the correct exclusion of complete AVB. This study probably opens gates to future directions in the evaluation of the conductance disturbances after percutaneous PmVSD closure.

## Figures and Tables

**Table 1:** Demographic characteristics of the patients.

Demographics	All patients
*N*	119

Age (yrs)	5 (0.8–54)

Female (%)	62 (52.5)

Weight (kg)	18 (6.5–87)

Height (cm)	110 (70–170)

VSD size (mm)	4.8 (2–13)

Occluder devices	
ADO I (*n*, %)	(55, 46.6%)
5/4 mm device	1
6/4 mm device	7
8/6 mm device	19
10/8 mm device	22
12/10 mm device	5
16/14 mm device	1
ADO II (*n*, %)	(51, 43.2)
3/4 device	2
4/4 device	7
5/4 device	9
5/6 device	2
6/4 device	26
6/6 device	5
AVP II (*n*, %)	(12, 10.2)
6 mm device	2
8 mm device	8
12 mm device	2

Vascular approach	
Prograde (%)	62 (52.1)
Retrograde (%)	58 (47.9)

*Success rate (n, %)*	(117, 98.3)

ADO: Amplatzer duct occluder. AVP: Amplatzer vascular plug.

## Data Availability

Previously reported manuscripts were used to support this study and are available at DOI: 10.1111/j.1540-8183.2005.04051.x; DOI: 10.4236/wjcd.2013.32035; DOI: 10.1016/j.amjcard.2015.10.010; DOI: 10.1002/ccd.23074; DOI: 10.1016/j.jacc.2012.08.1004; DOI: 10.5812/ijp.386; and DOI: 10.1007/s00246-016-1553-x. These prior studies are cited at relevant places within the text (discussion section) as references [19, 20, 22–26].

## Abbreviations

VSD: Ventricular septal defect.
ADO: Amplatzer duct occluder.
AVP: Amplatzer vascular plug.
PmVSD: Perimembranous ventricular septal defect.
PDA: Patent ductus arteriosus.
ASD: Atrial septal defect.
AMVO: Amplatzer muscular ventricular occluder.
APMVSDO: Amplatzer perimembranous ventricular septal defect occluder.
TEE: Transesophageal echocardiography.
TTE: Transthoracic echocardiography.

## References

[1] Mavroudis C., Backer C. L., Jacobs J. P. (2003). Ventricular septal defect. *Pediatric Cardiac Surgery*.

[2] Bol-Raap G., Weerheim J., Kappetein A. P., Witsenburg M., Bogers A. J. J. C. (2003). Follow-up after surgical closure of congenital ventricular septal defect. *European Journal of Cardio-Thoracic Surgery*.

[3] Arora R., Trehan V., Kumar A., Kalra G. S., Nigam M. (2003). Transcatheter closure of congenital ventricular septal defects: experience with various devices. *Journal of Interventional Cardiology*.

[4] Lock J. E., Block P. C., McKay R. G., Baim D. S., Keane J. F. (1988). Transcatheter closure of ventricular septal defects. *Circulation*.

[5] Sideris E. B., Walsh K. P., Haddad J. L., Chen C. R., Ren S. G., Kulkarni H. (1997). Occlusion of congenital ventricular septal defects by the buttoned device. “Buttoned device” clinical trials international register. *Heart*.

[6] Janorkar S., Goh T., Wilkinson J. (1999). Transcatheter closure of ventricular septal defects using the rashkind device: initial experience. *Catheterization and Cardiovascular Interventions*.

[7] Kalra G. S., Verma P. K., Dhall A., Singh S., Arora R. (1999). Transcatheter device closure of ventricular septal defects: immediate results and intermediate-term follow-up. *American Heart Journal*.

[8] Ramaciotti C., Keren A., Silverman N. H. (1986). Importance of (perimembranous) ventricular septal aneurysms in the natural history of isolated perimembranous ventricular septal defect. *The American Journal of Cardiology*.

[9] Pedra C. A., Pedra S. R., Esteves C. A. (2004). Percutaneous closure of perimembranous ventricular septal defects with the Amplatzer device: technical and morphological considerations. *Catheterization and Cardiovascular Interventions*.

[10] Thanopoulos B. D., Tsaousis G. S., Karanasios E. (2003). Transcatheter closure of perimembranous ventricular septal defects with the Amplatzer asymmetric ventricular septal defect occluder: preliminary experience in children. *Heart*.

[11] Hijazi Z. M., Hakim F., Haweleh A. A. (2002). Catheter closure of perimembranous ventricular septal defects using the new Amplatzer membranous VSD occluder: initial clinical experience. *Catheterization and Cardiovascular Interventions*.

[12] Butera G., Carminati M., Chessa M. (2007). Transcatheter closure of perimembranous ventricular septal defects: early and long-term results. *Yearbook of Cardiology*.

[13] Holzer R., de Giovanni J., Walsh K. P. (2006). Transcatheter closure of perimembranous ventricular septal defects using the amplatzer membranous VSD occluder: immediate and midterm results of an international registry. *Catheterization and Cardiovascular Interventions*.

[14] Hieu N. L. Transcatheter closure of ventricular septal defects with new devices in Vietnam.

[15] Anderson R. H., Becker A. E., Tynan M. (1986). Description of ventricular septal defects—or how long is a piece of string?. *International Journal of Cardiology*.

[16] Amin Z., Gu X., Berry J. M. (1999). New device for closure of muscular ventricular septal defects in a canine model. *Circulation*.

[17] Thanopoulos B. D., Tsaousis G. S., Konstadopoulou G. N. (1999). Transcatheter closure of muscular ventricular septal defects with the amplatzer ventricular septal defect occluder: initial clinical applications in children. *Journal of the American College of Cardiology*.

[18] Carminati M., Butera G., Chessa M., Drago M., Negura D., Piazza L. (2005). Transcatheter closure of congenital ventricular septal defect with amplatzer septal occluders. *The American Journal of Cardiology*.

[19] Behnke I. M., Le T. P., Waldecker B., Akintuerk H., Valeske K., Schranz D. (2005). Percutaneous closure of congenital and acquired ventricular septal defects-considerations on selection of the occlusion device. *Journal of Interventional Cardiology*.

[20] Dilawar M., Ahmad Z. (2013). Safety and efficacy of Amplatzer duct occluder for percutaneous closure of ventricular septal defects with tunnel shape aneurysm: medium term follow up. *World Journal of Cardiovascular Diseases*.

[21] Diab K. A., Cao Q.-L., Mora B., Hijazi Z. M. (2007). Device closure of muscular ventricular septal defects in infants less than one year of age using the amplatzer devices: feasibility and outcome. *Catheterization and Cardiovascular Interventions*.

[22] Ebeid M. R., Batlivala S. P., Salazar J. D. (2016). Percutaneous closure of perimembranous ventricular septal defects using the second-generation amplatzer vascular occluders. *The American Journal of Cardiology*.

[23] El Said H. G., Bratincsak A., Gordon B. M., Moore J. W. (2012). Closure of perimembranous ventricular septal defects with aneurysmal tissue using the amplazter duct occluder I: lessons learned and medium term follow up. *Catheterization and Cardiovascular Interventions*.

[24] Koneti N. R., Sreeram N., Penumatsa R. R., Arramraj S. K., Karunakar V., Trieschmann U. (2012). Transcatheter retrograde closure of perimembranous ventricular septal defects in children with the amplatzer duct occluder II device. *Journal of the American College of Cardiology*.

[25] Ghaderian M., Merajie M., Mortezaeian H., Aarabi M., Mohammad Y., Shah Mohammadi A. (2015). Efficacy and safety of using amplatzer ductal occluder for transcatheter closure of perimembranous ventricular septal defect in pediatrics. *Iranian Journal of Pediatrics*.

[26] El-Sisi A., Sobhy R., Jaccoub V., Hamza H. (2017). Perimembranous ventricular septal defect device closure: choosing between amplatzer duct occluder I and II. *Pediatric Cardiology*.

[27] Hua N., Aquino P., Owada C. Y. (2016). Transcatheter closure of perimembranous ventricular septal defects with the amplatzer vascular plug-II. *Cardiology in the Young*.

[28] Kanaan M., Ewert P., Berger F., Assa S., Schubert S. (2015). Follow-up of patients with interventional closure of ventricular septal defects with amplatzer duct occluder II. *Pediatric Cardiology*.

[29] Zhao L.-J., Han B., Zhang J.-J., Yi Y.-C., Jiang D.-D., Lyu J.-L. (2017). Transcatheter closure of congenital perimembranous ventricular septal defect using the Amplatzer duct occluder 2. *Cardiology in the Young*.

[30] Knop M. T., Litwin L., Szkutnik M., Białkowski J., Galeczka M., Fiszer R. (2018). Percutaneous closure of perimembranous and postsurgical ventricular septal defects with amplatzer duct occluder II additional sizes in paediatric patients-case series. *Advances in Interventional Cardiology*.

